# Acute renal failure due to severe bilateral ureteropelvic junction obstruction treated by urinary drainage in a 2‐year‐old infant

**DOI:** 10.1002/iju5.12397

**Published:** 2021-11-11

**Authors:** Kazuto Suda, Hideaki Nakajima, Toshihiro Yanai

**Affiliations:** ^1^ Departments of Pediatric Surgery Ibaraki Children’s Hospital Mito Ibaraki Japan; ^2^ Department of Pediatric Urology Ibaraki Children’s Hospital Mito Ibaraki Japan

**Keywords:** emergent drainage, hydronephrosis, renal failure, ureteropelvic junction obstruction, ureterovesical junction stenosis

## Abstract

**Introduction:**

Conscientious follow‐up is essential for bilateral grade 4 hydronephrosis with ureteropelvic junction obstruction to ensure optimal surgical timing. We have reported a case of a male infant who required emergent urinary drainage due to severe bilateral ureteropelvic junction obstruction‐derived acute renal failure.

**Case presentation:**

Bilateral grade 4 hydronephrosis was diagnosed in a male neonate. Vesicoureteral reflux was ruled out. Two years after the initial diagnosis, he developed acute renal failure and underwent bilateral emergent urinary drainage, followed by multiple urinary tract reconstructions against left ureterovesical junction stenosis and bilateral ureteropelvic junction obstruction. The postoperative renogram demonstrated a bilateral nonobstructive pattern.

**Conclusion:**

Bilateral emergency drainage for acute renal failure was successful without hemodialysis. Unilateral drainage or pyeloplasty should be planned early for bilateral grade 4 hydronephrosis with ureteropelvic junction obstruction to avoid lethal events if the obstruction pattern with decreased split renal function is <40% or if it is symptomatic.

**Abbreviations & Acronyms**
ARF: acute renal failure
BUN: blood urea nitrogen
BUN: blood urea nitrogen
HN: hydronephrosis
MAG3: mercaptoacetyltriglycine
SFU: Society of Fetal Urology
SRF: split renal function
UPJO: ureteropelvic junction obstruction
UTI: urinary tract infection
UVJS: ureterovesical junction stenosis

**Keynote message**

The risk of acute renal failure risk, although rare, must be considered in patients with bilateral grade 4 hydronephrosis with ureteropelvic junction obstruction. Unilateral drainage or pyeloplasty should be planned early for grade 4 hydronephrosis with ureteropelvic junction obstruction if the obstruction pattern with decreased split renal function is <40% or if it is symptomatic. Unilateral emergent drainage is necessary, at least in cases of acute renal failure, and bilateral procedures might be preferable to preserve renal function.

## Introduction

The duration of follow‐up and proper timing of surgery for UPJO, particularly in cases of bilateral high‐grade HN,^1^, ^2^, ^3^, ^4^ is critical as long‐term high‐grade HN may affect renal functional outcomes. Decreased renal function on renography, UTI, and colic pain could be indications for urinary drainage or pyeloplasty during conservative management.^5^, ^6^, ^7^ However, there are no reports regarding emergent treatment for patients with ARF and their prognosis. Here, we have presented a rare case of ARF due to bilateral grade 4 HN with UPJO that was treated with emergent drainage and multistage urinary tract reconstruction.

## Case report

**Fig. 1:**
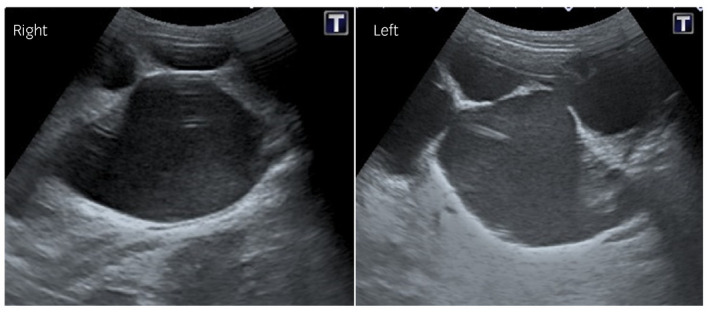
Intraoperative ultrasonography showing severe right and left dilatation of the renal pelvis and calyx with thinning parenchyma.

**Fig. 2:**
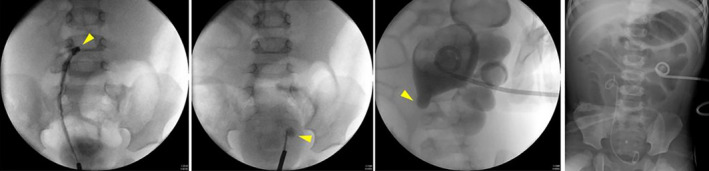
Left to right panel: Inflow jet into the right pelvis is seen in the retrograde ureteral contrast study (arrowhead). Insertion of ureteral stent into the left ureteral orifice is not possible during retrograde ureterography (arrowhead). Pyelography through the left nephrostomy indicating an obstruction (arrowhead). The left percutaneous nephrostomy and the right ureteral stent are seen in post‐drainage X‐ray imaging.

**Fig. 3:**
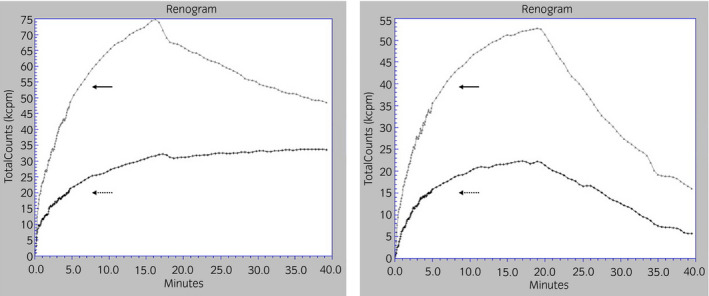
Left panel: Initial mercaptoacetyltriglycine scintigraphy immediately after emergent drainage shows an obstructive pattern on the left side and delayed excretion on the right side (right: arrow, left: dotted arrow). Right panel: Another scintigraphy 6 months after the last reconstructive surgery reveals bilateral release from the urinary tract obstruction pattern (right: arrow, left: dotted arrow).

Bilateral HN was detected in a male fetus on ultrasonography by a previous doctor, and initial ultrasonography immediately after birth revealed bilateral SFU grade 4 HN. Vesicoureteral reflux was ruled out by voiding cystourethrography. Dimercapto succinic acid scintigraphy revealed right‐dominant (67%) SRF at 1 and 2 years old. Two years after the initial diagnosis, he presented with a sudden onset of high fever and oliguria. He was transferred to our hospital while on glucose‐insulin therapy since a blood test suggested ARF with hyperkalemia (BUN level, 75 mg/dL; creatinine level, 5.6 mg/dL; potassium level, 7.2 mEq/L). Another ultrasound revealed bilateral SFU grade 4 HN (Fig. 1), prompting emergent drainage. A 4.7‐Fr ureteral stent was inserted against the right UPJO via retrograde ureterography (Fig. 2). As a ureterographic stent could not be inserted in the left ureter due to UVJS (Fig. 2), percutaneous nephrostomy using an 8.3‐Fr catheter was performed in the left kidney, as indicated for left UPJO by antegrade pyelography (Fig. 2). The bilateral HN was relieved immediately after emergent drainage (Fig. 2). Within 48 h of drainage, the levels of serum BUN, creatinine, and potassium improved to 11 mg/dL, 0.36 mg/dL, and 3.6 mEq/L, respectively, which were to the normal range for children aged 2 years. Urine culture tests at admission did not indicate bacterial growth. Rotavirus, norovirus enteritis, or renal calculi were not detected. Renal MAG3 scintigraphy demonstrated right‐dominant SRF (68%). An obstructive pattern on the left side and delayed excretion on the right side were observed. The obstructive pattern on the right side was likely masked by the ureteral stent (Fig. 3).

**Fig. 4:**
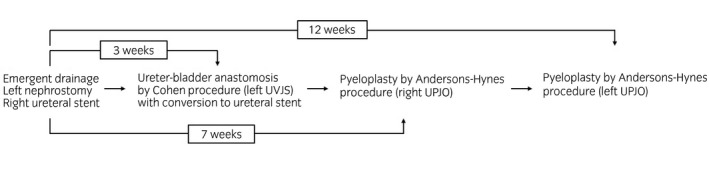
Timetable showing a multi‐planned surgical intervention for urinary tract construction. Cohen procedure against left ureterovesical junction stenosis and pyeloplasty for the right and left ureteropelvic junction obstruction performed at 3, 7, and 12 weeks, respectively, after emergent drainage.

After emergent drainage, the following multistage operations were performed: (i) the Cohen procedure on the left UVJS and (ii) conventional pyeloplasty on the bilateral UPJO after 3, 7, and 12 weeks (Fig. 4). During the Cohen procedure, a 1.5‐cm ureteral stenotic region was removed, and a ureter–bladder anastomosis was created. Meanwhile, the nephrostomy was switched to a ureteral stent. Histopathological analysis identified a fibrotic scar in the resected tissue at the left ureterovesical junction. Abnormal bending without aberrant vessels or a high‐inserting ureter was observed in both ureteropelvic junctions during pyeloplasty. The Andersons–Hynes procedure with 4.7‐Fr stenting was performed bilaterally. Luminal narrowing, muscular hypertrophy, ureteral polyps, and fibrotic scarring were not detected in either resected specimen histopathologically. Both ureteral stents were removed 2 months after left pyeloplasty. The patient’s postoperative course was uneventful, and MAG3 scintigraphy 6 months after the last reconstructive surgery revealed right‐dominant (70%) renal function and a bilateral non‐obstructive pattern with T1/2 < 15 min (Fig. 3).

## Discussion

This is an extremely rare case of severe bilateral UPJO that was successfully treated using urinary drainage without hemodialysis against ARF in the pediatric field. Aoun et al.^8^ reported a solitary kidney case presenting with ARF due to UPJO which was treated with peritoneal dialysis. It has been theorized that ureteral edema or debris accumulation caused by UTI or high fever‐derived dehydration can promote progressive obstruction. Furthermore, norovirus‐ or rotavirus enteritis‐related renal calculi can block urine flow in the upper urinary tract.^9^ Our case did not present with UTI or renal calculus‐related evidence. Nonspecific viral fever‐related dehydration and debris accumulation in the ureteropelvic junction might have triggered disease onset.

Unilateral urinary drainage is required at least for the decreased renal function side in severe bilateral UPJO because spontaneous contralateral hydronephrosis recovery can be expected by decreasing the relative urine load after unilateral release of obstruction.^1^ We performed bilateral drainage because we believed that acute exacerbation on both sides of the obstruction caused ARF. Furthermore, we considered persistent renal damage without drainage.

A ureteral stent is preferable to a percutaneous nephrostomy if cystoscopy can be performed to avoid complicated handling and risk of accidental tube removal. Thus, we planned to treat the UVJS first to switch the left nephrostomy to a ureteral stent perioperatively. A ureteral stent for UPJO enables an enlarged ureter by promoting urine inflow and easy ureter identification with the stent during pyeloplasty.

Our patient was conservatively observed until he developed ARF at 2 years old. An appropriate surgical intervention strategy for patients with severe bilateral UPJO is critical, as long‐term high‐grade HN may affect renal functional outcomes. Kohno et al. recommend a close follow‐up every 1–3 months with further evaluation by renography for the conservative management of severe bilateral UPJO.^7^ The indication of drainage or pyeloplasty is determined if the bilateral obstructive pattern with decreased SRF is <40%, grade 4 HN is observed for 3 years, or if the patient is symptomatic during the neonatal period. In our case, urinary drainage or pyeloplasty should have been planned early, at least unilaterally before the onset of ARF because of decreased SRF in the left kidney to avoid lethal complications and preserve renal function.

## Conclusions

Unilateral drainage or pyeloplasty should be planned early for severe bilateral UPJO if the obstruction pattern with decreased SRF is <40% or if it is symptomatic. Further, unilateral emergent drainage is necessary, at least in cases of ARF, and bilateral procedures might be preferable to preserve renal function.

## Author Contribution

Kazuto Suda: Data curation; Investigation; Project administration; Resources; Writing‐original draft; Writing‐review & editing. Hideaki Nakajima: Data curation; Investigation; Resources. Toshihiro Yanai: Conceptualization; Project administration; Supervision; Writing‐review & editing.

## Conflict of interest

The authors declare no conflict of interest.

## Approval of the research protocol by an institutional reviewer board

Not applicable.

## Informed consent

Not applicable.

## Registry and registration no. of the study/trial

Not applicable.
